# Development and Validation of Near-Infrared Methods for the Quantitation of Caffeine, Epigallocatechin-3-gallate, and Moisture in Green Tea Production

**DOI:** 10.1155/2021/9563162

**Published:** 2021-11-15

**Authors:** Shengsheng Zhang, Yamin Zuo, Qing Wu, Jiao Wang, Lin Ban, Huili Yang, Zhiwen Bai

**Affiliations:** ^1^Guizhou Key Laboratory for Information System of Mountainous Areas and Protection of Ecological Environment, Guizhou Normal University, 116 Baoshan North Rd, Guiyang, Guizhou 550001, China; ^2^School of Basic Medical Sciences, Hubei Key Laboratory of Wudang Local Chinese Medicine Research, Hubei University of Medicine, 30 Renmin South Rd, Shiyan, Hubei 442000, China; ^3^The Guizhou Gui Tea (Group) Co. Ltd, Huaxi District, Guiyang, Guizhou 550001, China

## Abstract

The quality of tea leaves (e.g., their color, appearance, and taste) can be directly influenced by the tea production process, which is closely connected with the content of a number of chemical components formed during the production of the tea leaves. However, the production process is now controlled by people's experience, making its quality significantly different. NIRS is a time-saving, cost-saving, and nondestructive method. Therefore, it is necessary to introduce NIRS technology into the quality control of the tea production process. In this study, a quantitative analysis model of caffeine, epigallocatechin-3-gallate (EGCG), and moisture content was established by near-infrared spectroscopy (NIRS) which was united simultaneously with partial least squares (PLSR) for online process monitoring of tea production. The model parameters show that the established model has fine robustness and outstanding measuring accuracy. Then, the feasibility of the established method is verified by the traditional method. Through the verification of the precision of the instrument and the stability of the sample, it is clarified that the model can be further utilized to monitor tea product quality online in a productive process.

## 1. Introduction

**Figure 1:**
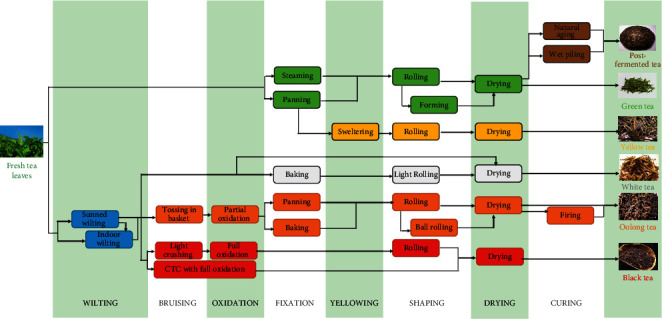
The production process of various teas.

Tea is made of tea buds and leaves of *Camellia sinensis*. It is known as the second largest drink in the world [1]. Its production process is composed of fixation, withering, rolling, fermentation, polling, drying, etc. The different kinds of tea (green, black, and white tea) are basically different in their production process (Figure 1) [2]. The two most best-selling categories are green (unfermented) tea and black (fully fermented) tea, accounting for 98% of the world's tea production and consumption approximately [3].

In 2015, the Intergovernmental Group for Tea, which was established by the Food and Agricultural Organization (FAO), outlined the current market conditions and intermediate-term market prospects for teas (until 2023). The world's green tea exports are expected to grow at an annual rate of 7.1% and reach 750981 tons by 2023. China is projected to dominate continuously, with exports of 458579 tons [4]. Tea quality is very essential for its market value, which is conventionally evaluated by skilled tea tasters. However, quality is determined by factors such as the color, appearance, and flavor of the tea. These factors are closely related to some changes in the content of certain chemical components, including catechins, moisture content, and caffeine, during the production of tea [5–8].

In this complex composition, catechin is considered to be the most healthy component of green tea, accounting for about 25% of the dry weight of fresh tea leaves [9, 10]. The composition of catechins in green tea varies with light variation, growing location, clonal variation, species, season, and altitude, and mainly with processing technology. EGCG, a substance with relatively high catechin content, is the main source of the astringency and bitterness of tea beverages and is a biologically active compound considered to be a significant qualitative factor in tea leaves [5, 11, 12].

Caffeine, which composes about 3% of the dry weight of tea leaves, is known for its stimulating properties and is an important quality factor in tea. Compared with the catechin in tea polyphenols, caffeine is very significant in improving the flavor of tea [13, 14].

**Figure 2:**
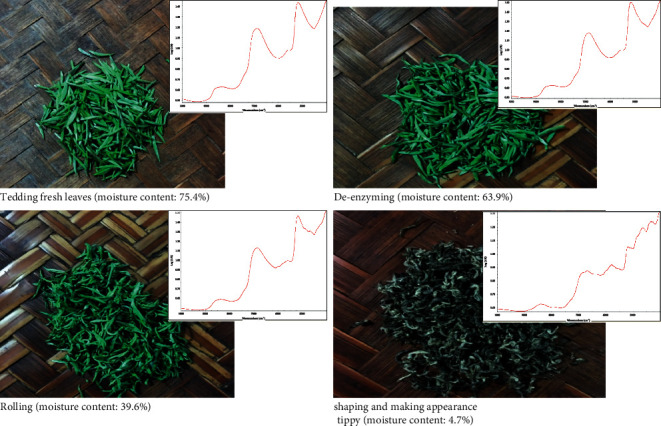
Moisture content changes in the tea production process.

The quality of tea also depends on its moisture content (Figure 2). Green tea is affected by moisture during transportation and storage. The affected green tea will produce a train of complex biochemical reactions, such as oxidation, polymerization, and degradation, resulting in poor tea quality [15, 16]. Water content is an important index to measure the quality of green tea, which has an important impact on the shape, color, aroma, and taste of green tea [17]. By controlling the moisture content, the inevitable deterioration caused by changes in the physicochemical properties of tea leaves can be avoided, and the sensory properties of tea leaves can be kept fresh and stable during long-term storage [18–22].

For the sake of improvement of the efficiency of the tea production process and development of high-quality tea products, it is very significant to establish a real-time and credible analytical method for the measurement of these chemical compounds in tea samples.

In recent years, NIRS, a powerful tool, has been widely used for a series of processes, including raw material testing, product quality control, and process monitoring [23–25]. NIRS is a simple, rapid, and nondestructive analytical technique. In addition, compared with other analysis techniques, it enables the analysis of complex matrices without manipulating samples, greatly shortening the analysis time, which has aroused the growing interest of the pharmaceutical industry. [26, 27]. So far, the application of NIRS in process monitoring has been widely reported. Zuo et al. [28] explored the potential applications of NIRS in monitoring the steaming process of *Gastrodiae rhizoma*. To confirm four chemical compositions and moisture changes in the process of steaming, about 10 laboratory-scale batches were utilized to establish quantitative models. Wu et al. [29] developed a pragmatic model enabling monitoring of the extraction process online in red peony by NIRS. The establishment of the models was utilized to monitor the online extraction process in real time. At the same time, considering the long-term application of the developed models, a model updating method was proposed. However, in tea studies, NIRS is only used to determine the content of chemical components in finished tea leaves, and few studies report that NIRS enabled monitoring of the tea production process [30–33]. In addition, traditional methodological validation was not used to check instrument accuracy and sample stability.

In this study, a method for the determination of moisture content and caffeine in green tea production was developed by using NIRS and partial least square regression. The root mean square error of prediction (RMSEP) and the coefficient of determination (*R*^2^) of the prediction set were used for evaluating the performance of the finished model. The traditional methodology was used to validate the performance of the model in terms of instrument precision and sample stability so that it could be monitored in real time during the production process. This controllable and high-efficiency production technology could be used in the future as a preliminary basis for large-scale production of high-quality tea.

## 2. Experimental Methods

### 2.1. Reagents and Materials

A total of 39 batches of green tea samples were supplied by Qiannan Meiyuan Trade Co., Ltd. (Guizhou, China). Reference substance of caffeine (batch No. wkq20041403) and EGCG (batch No. wkq17122008) was purchased from Sichuan Weiqi Biological Technology Co., Ltd. (Sichuan, China). Methanol and acetonitrile were purchased from Kemmo Chemical Reagent (Tianjin, China). Water was purified by using a Milli-Q water purification device (Millipore, USA). The other chemical reagents were all of analytical grade.

### 2.2. Preparation of Solutions

#### 2.2.1. Preparation of Reference Solutions

The appropriate amount of caffeine or EGCG was accurately weighed into two volumetric flasks of 10 mL each. Subsequently, 10 mL of methanol was added to each flask, and the mixtures were mixed by ultrasonication to obtain two reference stock solutions of caffeine or EGCG with concentrations of 3.47 mg/mL and 5.05 mg/mL, respectively.

Then, 1 mL of the reference stock solution was pipetted into a 10 mL volumetric flask. Methanol was added to the scale line and sonicated and mixed to obtain reference solutions of 0.347 mg/mL and 0.505 mg/mL for caffeine and EGCG, respectively.

#### 2.2.2. Preparation of Sample Solutions

Different batches of tea leaves were accurately weighed in a 50 mL conical flask, then 25 mL of 70% methanol solution was added, capped, and weighed, the ultrasonic extraction was carried out for 30 minutes (power 500 W and frequency 45 kHz), the conical flask was allowed to cool to room temperature, the leaves were weighted again, the reduced weight was supplemented with 70% methanol, the solution was mixed thoroughly and allowed to stand for 10 minutes, and the supernatant was passed through a 0.22 *μ*m filter membrane to obtain the sample solution.

### 2.3. Chromatographic Conditions

**Figure 3:**
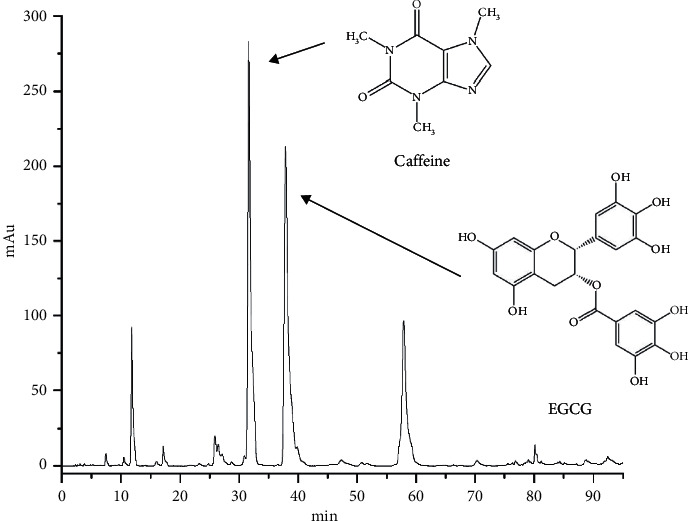
A typical HPLC chromatogram and the chemical structures of caffeine and EGCG.

The chromatographic conditions are as follows: chromatographic column, Diamonsil C_18_ column (4.6 mm × 250 mm, 5 *μ*m); flow rate, 1.0 mL/min; injected volume, 10 *μ*L; column temperature, 30°C; detection wavelength, 278 nm; and mobile phase, (A) water/acetic acid (1000 : 1, v/v) and (B) acetonitrile. The gradient used was as follows: 0–13 minutes, 6%–12% B; 13–23 minutes, 12%–18% B; 23–31 minutes, 18%–18% B; 31–36 minutes, 18%–21.5% B; 36–44 minutes, 21.5%–25% B; 44–54 minutes, 25%–63% B; and 54–70 minutes, 63%–70% B. The HPLC chromatogram and the structural formula of the substance to be tested are shown in Figure 3.

### 2.4. Determination of Moisture Content

A 5 g sample of tea was weighed into a bottle and subjected to heat treatment at 103 ± 2°C until it reached a constant weight. The weight lost from the sample expressed as a percentage represents its moisture content [3].

### 2.5. NIR Acquisition, Modeling, and Model Verification

#### 2.5.1. Acquisition Conditions

The ANTARIS II FT-NIR analyser was used for spectral acquisition during the entire tea production process. The acquisition process is as follows: detection wavelength range 4,000 to 10,000 cm^−1^ and resolution 8 cm^−1^. 5 ± 0.1 g of tea sample was put into the sample attachment (rotating cup), and then, the rotating cup was mounted on the NIR spectrometer and waited for detection. To eliminate background effects, three spectra of each sample were collected with air as a reference. Between each measurement, the cup with the tea sample was rotated by 120°. The average values of these three spectra were used in the following analysis. Taking into account that relative humidity and room temperature may affect the surface moisture of tea, relative humidity was maintained at 80% and room temperature was maintained at 25°C during the collection of spectra.

#### 2.5.2. Spectral Pretreatment

TQ Analyst software is used to analyse the spectrum. In addition to the improved prediction performance of the model, it is also necessary to transform the NIR spectra to remove irrelevant information and noise. Therefore, several different methods were used to convert the spectra. For the selection of the light range type, the multiplicative scattering correction (MSC) method was chosen because the light range could not be kept constant due to the particle size and homogeneity of the sample in this experiment; for the spectral shift problem, the first-derivative (FD) and second-derivative (SD) methods were chosen; for the filtering method of the spectra, the Savitzky–Golay (SG), no smoothing (Ns), and Norris derivative (ND) methods were chosen. For the quantification of these three compounds, PLS regression models were developed and employed, and their final performance was explored and compared systematically.

#### 2.5.3. Division of Calibration Sets and Test Sets

In order to ensure that the calibration set content range included the verification set content range, the contents of different batches were sorted in descending order. A total of 96 calibration sets and 60 test sets were selected.

#### 2.5.4. Model Verification

We followed the methods of Shi et al. [34]. The root mean square error between correction and prediction (RMSEC and RMSEP) and the correlation coefficient between correction and prediction (RC^2^ and RP^2^) were used as the evaluation indexes of the best quantitative model. A model is considered high performance if it has high *R*^2^ and low cross-validation root mean square error (RMSECV) and RMSEP values.

The quality of the model is not only based on the proper selection of modelling methods and spectral processing methods, as well as the evaluation of the model performance through *R*^2^, RMSEC, and RMSEP, but also the failure to consider the stability of the sample and the precision of the instrument will affect the establishment of the model. Therefore, it is necessary to use the traditional methodology for verification.

## 3. Results and Discussion

### 3.1. Optimization of the Extraction Efficiency of Components to Be Tested

**Figure 4:**
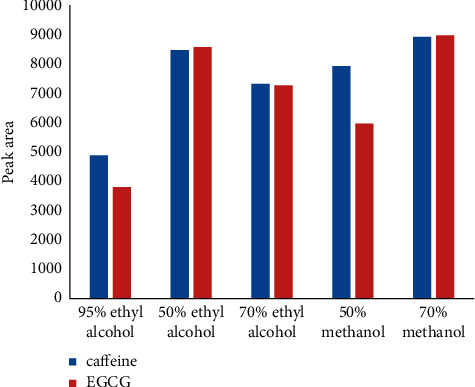
The peak area of two ingredients was compared under different extraction solvents.

Different solvents showed different extraction rates of the components to be tested. In this experiment, the extraction rates of 50% methanol, 70% methanol, 50% ethanol, 70% ethanol, and 95% ethanol solvents were investigated for the components to be tested in the samples. As shown in Figure 4, the peak areas of each component in HPLC were used to represent the extraction rates of different solvents. As can be seen in Figure 4, 70% methanol exhibited higher extraction efficiency.

### 3.2. Cluster Analysis of Tea Samples

**Figure 5:**
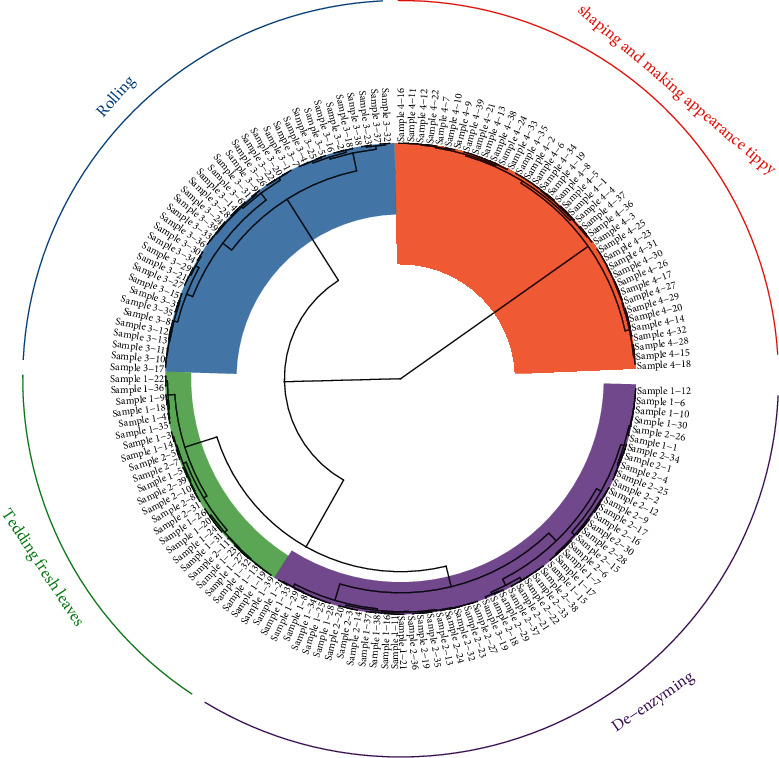
Cluster analysis of tea samples.

Cluster analysis was performed by analyzing the Euclidean distance between three relevant chemical components (moisture, caffeine, and EGCG) of the tea production process. As can be seen from Figure 5, the tea samples were grouped into 4 main categories, which basically correspond to the four key processes of tea production. This indicates that the contents of moisture, caffeine, and EGCG change with the tea production process, thus demonstrating the necessity of using NIR spectroscopy to monitor the changes of moisture, caffeine, and EGCG contents in the tea production process online.

### 3.3. Overview of Spectra

**Figure 6:**
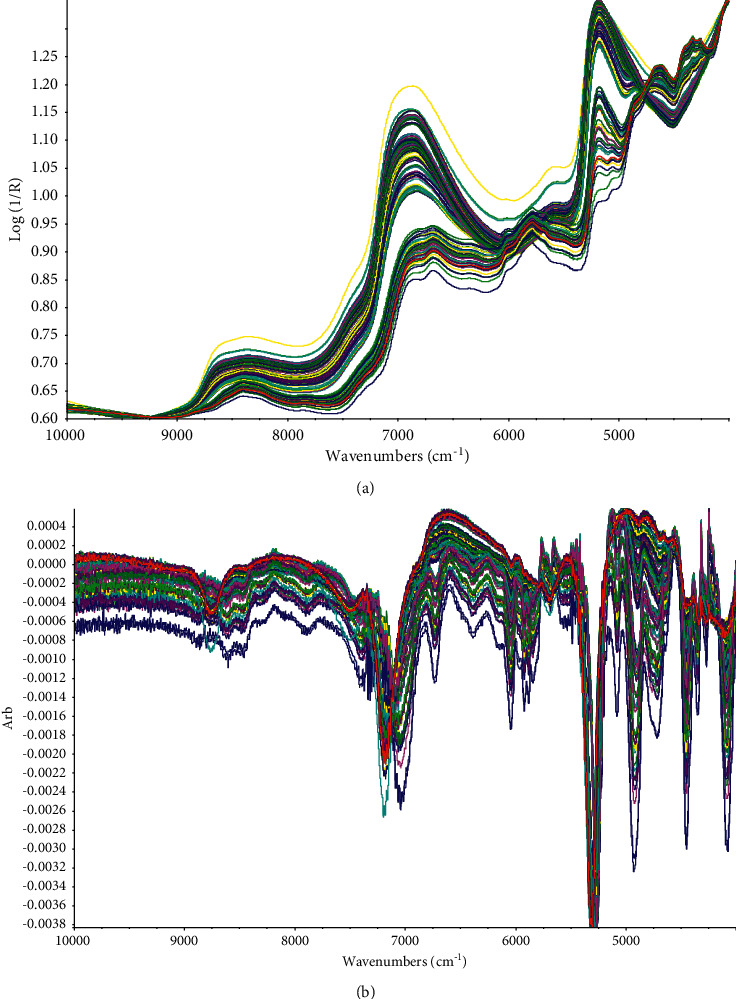
Original NIR spectra (a) and pretreated NIR spectra (b) of green tea samples.

Figures 6(a) and 6(b), respectively, present the spectra of the original data and after the first-order derivation. As shown in Figures 6(a) and 6(b), the peak values at 7000 cm^−1^ are in connection with the first stretching overtone of the moisture content O-H bond, and the peak values at 5155 cm^−1^ are in connection with the bending combination of the moisture content O-H bond. It can also be seen in Figure 6(b) that the peak at 5323 cm^−1^ in the spectrum is the second overtone vibration of the carbonyl group, followed by peaks at C-H (7212 cm^−1^), -CH_2_ (5742 cm^−1^), and -CH_3_ (5808 cm^−1^). The overtone, vibration, and stretching of these groups are all related to the chemical structure of the components to be tested, caffeine, EGCG, and moisture [13].

### 3.4. Reference Data Description

To build a PLS model, one hundred and fifty-six samples are selected in the experiment. In order to avoid a subset selection bias, we use the Kennard and Stone (K-S) algorithm, which covers the space in a uniform manner by maximizing the Euclidean distances between selected and remaining objects. Finally, we selected two-thirds of the total samples as training sets and the remaining samples as test sets, i.e., 96 spectra as a training set and the remaining 60 spectra as a test set. As seen from Tables 1 and 2, the scope of the training set contains the scope of the test set. Therefore, the choice of samples is appropriate.

### 3.5. Quantitative Analysis of the PLS Models

Due to the influence of various factors, the original spectrum contains not only its own near-infrared spectrum information but also other noise information. Therefore, it is necessary to preprocess spectral data by MSC + SD/FD + NS/SG/ND. In the processing of building PLS models, the “mean value” method is used to preprocess the data centrally. The performance of the final PLS models was evaluated by RMSECV, RMSEP, and *R*^2^.

It is well known that the two most critical parameters in the PLS modelling and optimisation process are the no. of PLS factors and the spectral preprocessing method. The optimum number of factors is determined by the minimum RMSECV.  Table 3 shows the results of the calibration model with variable spectral pretreatment methods to determine the contents of caffeine, moisture, and EGCG. Compared with other methods, for the caffeine, the lowest spectral pretreatment method of RMSECV was the combination of MSC + FD + N-D, which was 0.14580%, but there were 9 partial least squares factors in this model. Exorbitant PLS factors could contain particular information during modelling, resulting in a worse generalization performance of the PLS model. The “overfitting” of the model refers to this phenomenon in reality. Therefore, for the combination of MSC + FD + Ns as a spectral pretreatment method, the RMSECV value is 0.16092%; for the moisture content, the RMSECV value after MSC + FD + Ns combination spectral pretreatment is the lowest, which is 5.68517%; and for the EGCG, the RMSECV value after MSC + SD + N-d combination spectral pretreatment is the lowest, which is 0.31884%.  Table 4 shows the statistics of the best calibration model for the three indicators.

**Figure 7:**
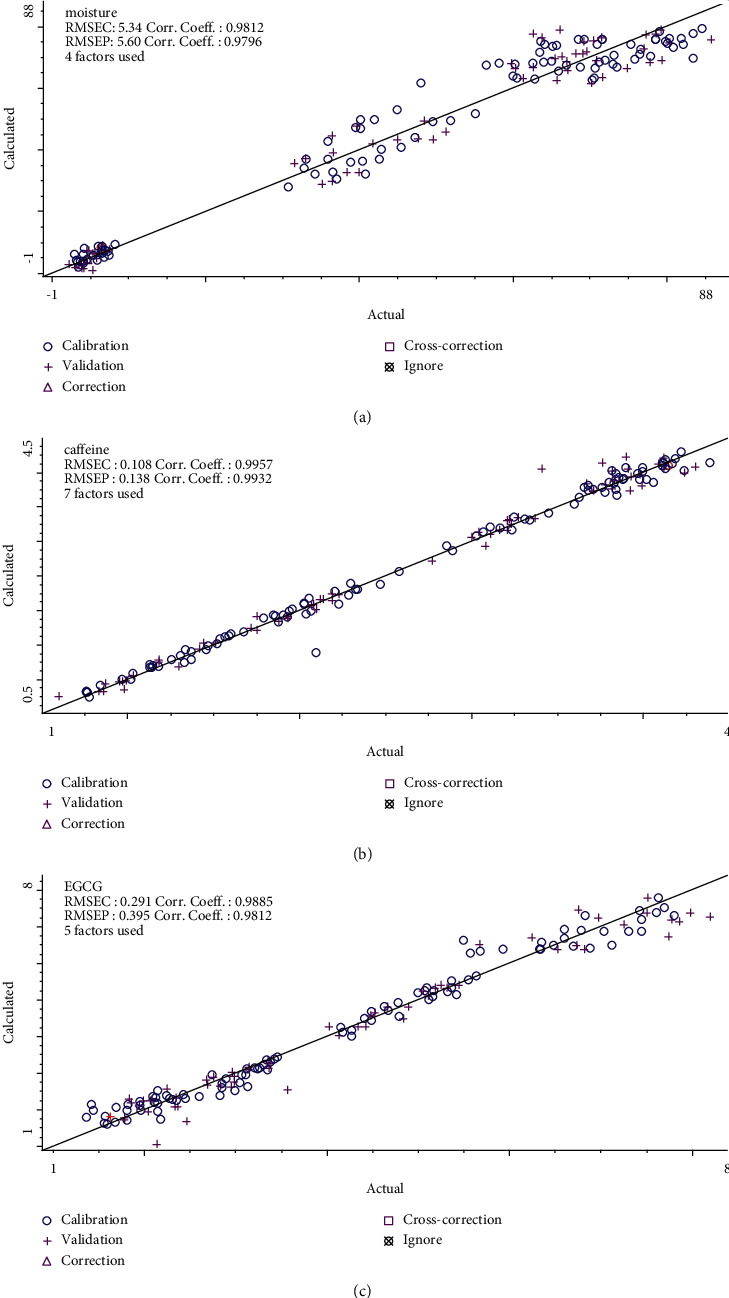
Prediction results of the three partial least squares models: (a) moisture, (b) caffeine, and (c) EGCG.

Scatter plots, Figures 7(a)–7(c), show the partial least squares quantitative models for the components to be tested, moisture, caffeine, and EGCG, respectively. The circles and plus signs in Figure 7 represent the data in the training group and the test group, respectively. Corresponding the training group data to the training group spectra and then using partial least squares to perform a linear fit, a good linear relationship was finally obtained for three components to be tested. As seen in Figure 7(a), the RMSEP value of water was 5.600, and the correlation coefficients were 0.9812 and 0.9796 for the training and test groups, respectively; as seen in Figure 7(b), the RMSEP value of caffeine was 0.138, and the correlation coefficients were 0.9957 and 0.9932 for the training and test groups, respectively; and as seen in Figure 7(c), the RMSEP value of EGCG was 0.395, and the correlation coefficients were 0.9885 and 0.9812 for the training and test groups, respectively.

**Figure 8:**
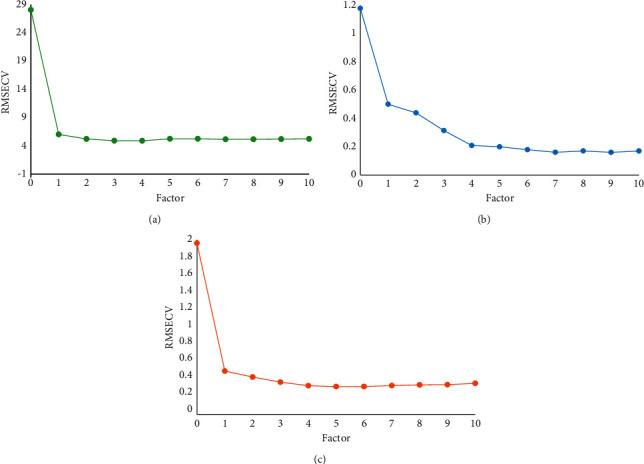
The variation of RMSECV with different principal factor numbers: (a) moisture, (b) caffeine, and (c) EGCG.

The predictive ability of a quantitative analytical model is directly related to the choice of the number of principal factors. Figures 8(a)–8(c) show the variation of RMSECV values with the number of principal factors for moisture, caffeine, and EGCG, respectively, of the substances to be measured. From Figure 8(a), it can be seen that the optimal number of principal factors for better predictive ability of the moisture analysis model is 4; from Figure 8(b), it can be seen that the optimal number of principal factors for better predictive ability of the caffeine analysis model is 7; and from Figure 8(c), it can be seen that the optimal number of principal factors for better predictive ability of the EGCG analysis model is 5.

### 3.6. Method Validation

After the establishment of the NIR quantitative model, it is necessary to evaluate the applicability of this model. Generally, the prediction results of the NIR method and the measurement results of the reference method are required to be statistically analyzed to judge the performance of the established model. To prove that this method is suitable for the corresponding testing requirements, based on the abovementioned evaluation indicators, such as *R*^2^, RMSEC, and RMSEP, this experiment intends to verify the model method in terms of linearity, accuracy, repeatability, intermediate precision, and robustness according to the guidelines of the International Conference on Harmonization (ICH) [35].

#### 3.6.1. Linearity

Linearity refers to the degree of the direct linear relationship between the detection results and the concentration (amount) of analyte in the test product, which is the basis of quantitative determination. Because the NIR spectrometric method is an indirect analysis method established by multiple linear regression, its linear study is different from the traditional analysis method. It can be evaluated by analyzing the relationship between the predicted value of NIR and the true value of the object to be measured.  Table 5 shows the linear verification results of the three quantitative models of caffeine, EGCG, and moisture content. The regression equations are *Y* = 0.9957*X* + 0.0223, *R*^2^ = 0.9957, *Y* = 0.9885*X* + 0.0944, *R*^2^ = 0.9885, *Y* = 0.9812X − 1.7039, and *R*^2^ = 0.9812. Among them, the gradient and intercept of the linear parameters directly affect the prediction error of the model. When the intercept is 0 and the slope is 1, the total error of the model is 0. Therefore, it can be concluded from the regression equation that all the NIR quantitative models established in this experiment have good linearity.

#### 3.6.2. Accuracy

The accuracy of the method is mainly evaluated by comparing the analysis of the NIR predicted values of the analytes to be measured with the reference values and a high degree of similarity of results in high accuracy. The accuracy was evaluated by a paired *t*-test between the NIR predicted value of the prediction set samples and the reference value, and the results are shown in Table 5. *t*-test results showed that the *P* values of the three models of caffeine, moisture content, and EGCG were all greater than 0.05, indicating that there was no significant difference between the predicted values of the three NIR quantitative models and the reference values, and the accuracy was good.

#### 3.6.3. Repeatability

According to the ICH guidelines, repeatability refers to the accuracy of the method in a shorter period of time and in the same operating environment. In this experiment, the same operator measured the NIR spectrum of the same sample 6 times on the same day to investigate the repeatability of the method. The results are shown in Table 5. The RSDs between the six predicted values of the three NIR quantitative models were 2.699%, 3.654%, and 1.459%, respectively, indicating good reproducibility.

#### 3.6.4. Intermediate Precision

Intermediate precision considers the effect of random factors on the analytical results in the same laboratory. Because of the differences in measurement results caused by different operators and operating times, in this experiment, two operators measured the same sample for three consecutive days to investigate the intermediate precision of the method. The results are shown in Tables 5 and 6. According to ANOVA, there was no significant difference between the results of NIR spectra measured by different operators and different operating times (*P* values were all greater than 0.05), indicating that the intermediate precision of the three NIR quantitative models was good.

#### 3.6.5. Robustness

The robustness examines the reliability of the NIR quantitative model in the normal course of application of the method. Through the paired *t*-test method, the contents of each component predicted by HPLC and NIR were compared, as shown in Table 7. Before the paired *t*-test, the F-test was used to compare the variance of the two methods to determine whether the difference was significant, and the results showed that the experimental statistic was lower than the critical level (for a significance level of 0.05). Therefore, it can be concluded that there is no significant difference in standard deviations between the two methods for the sample set.

## 4. Conclusions

This study assessed the practicability of NIR spectroscopy in the quality control of green tea. First, the credible NIR quantitative models of caffeine, moisture, and EGCG in the production process were established and their robustness was validated. Then, the feasibility of the proposed method was verified by the traditional method, and the precision of the instrument and the stability of the sample were verified. It was further demonstrated that the model could be applied in monitoring tea product quality online in the productive process. In addition, compared with the traditional HPLC method, this technology is fast and nondestructive and has obvious advantages, especially in terms of sample volume required, number of steps required, and time required (see Table 8), which is beneficial for automated plants to produce high-quality tea. In summary, the abovementioned study has shown that real-time and online monitoring of the green tea production process in an automated plant is feasible using NIR combined with PLS. However, further optimisation of the prediction model requires the collection of a larger sample to develop a more robust model.

## Figures and Tables

**Table 1:** Reference measurements and the number of samples in the training group.

Components	S.N. (%)	Range (%)	Mean (%)	S.D. (%)
Caffeine	96	0.60–4.39	2.66	1.16
Moisture content	96	2.96–84.70	47.09	27.90
EGCG	96	1.37–7.81	4.14	1.93

**Table 2:** Reference measurements and the number of samples in the testing group.

Components	S.N. (%)	Range (%)	Mean (%)	S.D. (%)
Caffeine	60	0.76–4.30	2.60	1.14
Moisture content	60	2.27–83.82	46.55	27.51
EGCG	60	1.63–7.20	4.25	2.00

**Table 3:** Results of different spectral pretreatment methods.

Parameters	Pretreatment	RMSEC	*Rc* ^2^	RMSECV	LVs
Caffeine	MSC + FD + Ns	0.108	0.9957	0.16092	7
	MSC + FD + SG	0.115	0.9951	0.16382	7
	MSC + FD + N-d	0.118	0.9948	0.14580	9
	MSC + SD + Ns	0.206	0.9840	0.39435	5
	MSC + SD + S-G	0.179	0.9880	0.28295	6
	MSC + SD + N-d	0.140	0.9927	0.18781	10

Moisture content	MSC + FD + Ns	5.34	0.9812	5.68517	4
	MSC + FD + S-G	5.37	0.9810	5.69519	4
	MSC + FD + N-d	5.62	0.9792	5.76396	2
	MSC + SD + Ns	5.78	0.9780	6.45729	6
	MSC + SD + S-G	5.97	0.9765	6.20606	3
	MSC + SD + N-d	5.75	0.9783	5.89641	2

EGCG	MSC + FD + Ns	0.357	0.9827	0.39602	4
	MSC + FD + S-G	0.318	0.9863	0.38964	5
	MSC + FD + N-d	0.285	0.9890	0.32740	6
	MSC + SD + Ns	0.338	0.9845	0.43708	4
	MSC + SD + S-G	0.358	0.9826	0.39088	3
	MSC + SD + N-d	0.291	0.9885	0.31884	5

**Table 4:** Data for the three indexes of the best calibration model indexes.

Components	Training groups			Test groups		Bias	LVs
	Rc^2^	RMSEC	RMSECV	Rp^2^	RMSEP		
Caffeine	0.9957	0.108	0.16092	0.9932	0.138	0.03	7
Moisture content	0.9812	5.340	5.68517	0.9796	5.600	0.45	4
EGCG	0.9885	0.291	0.31884	0.9812	0.395	0.07	5

**Table 5:** Validation results of the method.

Project	Describe	Caffeine	Moisture content	EGCG
Linearity	NIR prediction = *a* *∗* reference value + *b*	Training set	Training set	Training set
		*Y* = 0.9957*X* + 0.0223	*Y* = 0.9812*X* − 1.7039	*Y* = 0.9885*X* + 0.0944
		*R* ^2^ = 0.9957	*R* ^2^ = 0.9812	*R* ^2^ = 0.9885

Accuracy	The paired *t*-test between the NIR predicted value and the reference value of the sample in the validation set	Prediction set	Prediction set	Prediction set
		*t* = −1.441	*t* = −0.663	*t* = 0.505
		*p* = 0.162 (*α* = 0.05)	*p* = 0.513 (*α* = 0.05)	*p* = 0.618 (*α* = 0.05)

Repeatability	The same sample was measured 6 times on the same day by the same operator	Sample reference 3.45%	Sample reference 6.05%	Sample reference 7.15%
		SD = 0.095	SD = 0.202	SD = 0.107
		RSD = 2.699%	RSD = 3.654%	RSD = 1.459%

Intermediate precision	The same sample was measured by two operators for three consecutive days	Sample reference 3.45%	Sample reference 6.05%	Sample reference 7.15%
		SD = 0.029	SD = 0.179	SD = 0.039
		RSD = 0.835%	RSD = 2.968%	RSD = 0.554%

SD: standard deviation; RSD: relative standard deviation.

**Table 6:** Results from intermediate precision checks.

Caffeine (3.45%)			Moisture content (6.05%)			EGCG (7.15%)		
Project	Operator 1	Operator 2	Project	Operator 1	Operator 2	Project	Operator 1	Operator 2
Day 1	3.46	3.44	Day 1	6.07	5.97	Day 1	7.09	7.09
Day 2	3.46	3.47	Day 2	6.21	6.27	Day 2	7.18	7.17
Day 3	3.39	3.44	Day 3	5.87	5.83	Day 3	7.14	7.11
F-test	*P*=0.627		F-test	*P*=0.878		F-test	*P*=0.726	

**Table 7:** Results of robustness checks.

Components	Method	No. of samples	Mean	Variance	*P* value	
					F-test	*t*-test
Caffeine	HPLC	60	2.69	1.329	0.832	0.688
	NIR	60	2.63	1.364		

Moisture content	Lamdry	60	46.51	769.770	0.989	0.984
	NIR	60	46.58	786.460		

EGCG	HPLC	60	4.27	4.069	0.895	0.812
	NIR	60	4.21	3.887		

**Table 8:** Comparison of NIR and HPLC methods.

Item	NIR	HPLC
Time required	3 min	100 min
Number of steps required	2	>10
Sample volume required	0 g	0.5 g

## Data Availability

The data used to support the results of this study are consistent with the data in this paper. Any further information is available from the authors upon request.

## Supplementary Materials

Supplementary Materials Graphical abstract is included in the supplementary file.
