# Supplementary figures and images for: Development and Validation of Near-Infrared Methods for the Quantitation of Caffeine, Epigallocatechin-3-gallate, and Moisture in Green Tea Production
Source: J Anal Methods Chem. 2021 Nov 15;2021:9563162. doi: 10.1155/2021/9563162 (PMC8608528; doi:10.1155/2021/9563162)

**Graphical Abstract:**

**
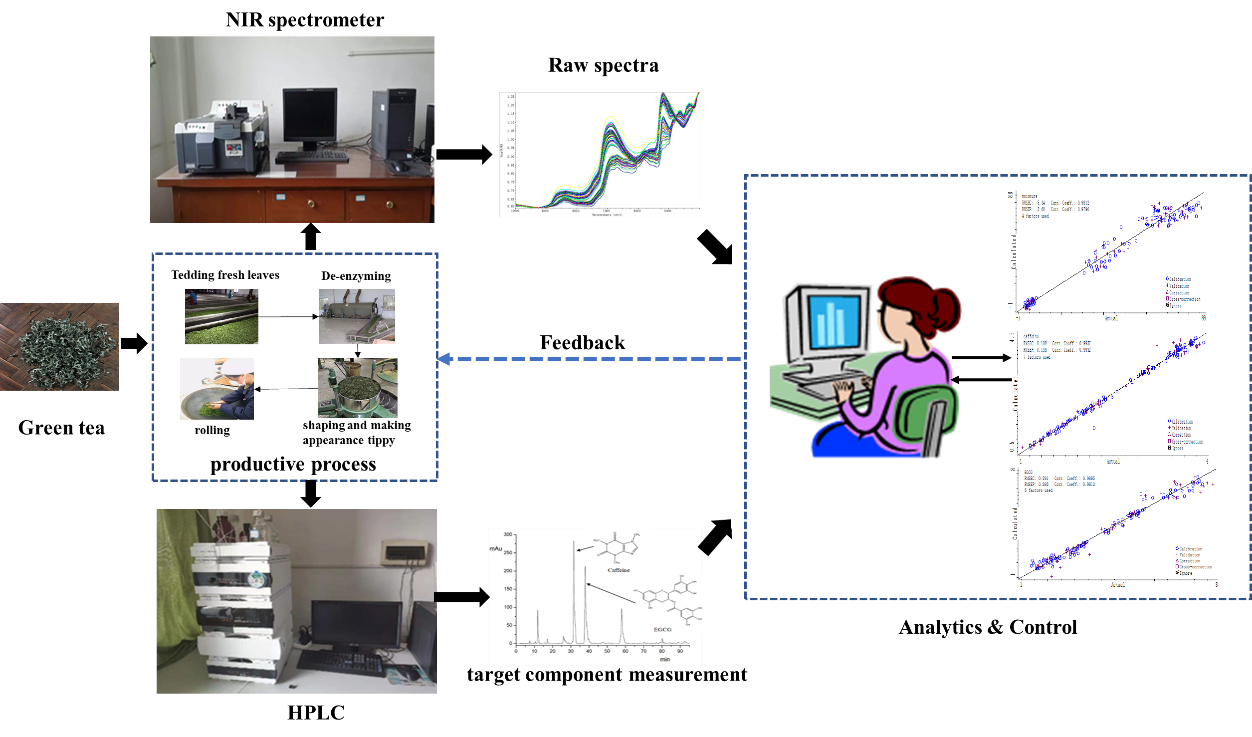
**

Supplement: Supplementary Materials — Graphical abstract is included in the supplementary file. [file 9563162.f1.docx]
